# A Review of Studies Leveraging Multimodal TMS-fMRI Applications in the Pathophysiology and Treatment of Schizophrenia

**DOI:** 10.3389/fnhum.2021.662976

**Published:** 2021-08-02

**Authors:** Sachin Pradeep Baliga, Urvakhsh Meherwan Mehta

**Affiliations:** ^1^Department of Psychiatry, TN Medical College and BYL Nair Charitable Hospital, Mumbai, India; ^2^Department of Psychiatry, National Institute of Mental Health and Neurosciences, Bengaluru, India

**Keywords:** brain connectivity, concurrent TMS/fMRI, simultaneous TMS-fMRI, psychosis, neuroplasticity, treatment resistance, causal inferences

## Abstract

The current review provides an overview of the existing literature on multimodal transcranial magnetic stimulation, and functional magnetic resonance imaging (TMS/fMRI) studies in individuals with schizophrenia and discusses potential future avenues related to the same. Multimodal studies investigating pathophysiology have explored the role of abnormal thalamic reactivity and have provided further evidence supporting the hypothesis of schizophrenia as a disorder of aberrant connectivity and cortical plasticity. Among studies examining treatment, low-frequency rTMS for the management of persistent auditory verbal hallucinations (AVH) was the most studied. While multimodal TMS/fMRI studies have provided evidence of involvement of local speech-related and distal networks on stimulation of the left temporoparietal cortex, current evidence does not suggest the superiority of fMRI based neuronavigation over conventional methods or of active rTMS over sham for treatment of AVH. Apart from these, preliminary findings suggest a role of rTMS in treating deficits in neurocognition, social cognition, and self-agency. However, most of these studies have only examined medication-resistant symptoms and have methodological concerns arising from small sample sizes and short treatment protocols. That being said, combining TMS with fMRI appears to be a promising approach toward elucidating the pathophysiology of schizophrenia and could also open up a possibility toward developing personalized treatment for its persistent and debilitating symptoms.

## Introduction

Schizophrenia is a severe mental illness characterized by positive (such as delusions, hallucinations), negative (anhedonia, asociality), cognitive (such as working memory deficits) symptom clusters. It has a life-time prevalence of around 1% and typically begins in late adolescence or early adulthood, leading to substantial disability, morbidity and mortality. While the exact pathophysiology of the illness remains elusive, schizophrenia is generally considered to be caused by a combination of genetic liability and environmental influences.

Current pharmacological strategies primarily focus on improving positive symptoms, with little or no effect on the negative and cognitive symptoms. Furthermore, the medications are effective in 1 only 50% of the cases, thus creating a need for newer strategies to target not only resistant positive symptoms, but also the other symptom clusters (de Araújo et al., 2012). Transcranial magnetic stimulation (TMS) is a neuromodulatory technique that acts via electromagnetic induction to generate an electric current in the superficial layers of the cortex. Single or paired-pulse TMS can be used as a neurophysiological probe to understand brain functions (Polanía et al., 2018). With these paradigms, TMS can have an excellent temporal resolution to the order of milliseconds (Bolognini and Ro, 2010). Further, when given repetitively in trains, rTMS can have differential effects by causing excitatory or inhibitory changes depending on the stimulation pattern and the cortical state (Wagner et al., 2007). These perturbation effects can cause plastic changes lasting 30–45 min and can be used to enhance or disrupt the underlying cortical networks. TMS over a target area causes effects in the underlying target areas and remote anatomically and functionally interconnected regions. Hence, TMS has limited spatial resolution when used on its own unless combined with an imaging modality (Wagner et al., 2007).

In contrast, functional magnetic resonance imaging (fMRI) offers the advantage of having a high spatial resolution. When combined with TMS as a neurophysiological probe, fMRI can be used to confirm the findings of ‘virtual lesions’ created using TMS (Pascual-Leone et al., 2000). Similarly, we can measure TMS-induced disruption of one node in a brain network on other distant nodes to yield brain connectivity metrics (Wagner et al., 2007). While fMRI has a weaker temporal resolution than EEG, it has an added advantage due to its ability to detect and monitor activity changes across larger and deeper areas such as subcortical structures (Siebner et al., 2009).

Electrophysiological and neurobiological research in the last two decades has demonstrated schizophrenia to be a disconnection syndrome involving widespread neuronal networks (Maran et al., 2016; Li et al., 2019; Mehta et al., 2019). However, these studies were based on individual applications of investigational techniques and were primarily correlational. Combining existing investigational techniques allows us to overcome their individual shortcomings and pave way for better understanding of pathophysiology of psychiatric illnesses. For example, combining TMS with EEG (TMS/EEG) can allow for simultaneous perturbation and measurement of neurophysiological correlates of cortical functioning in schizophrenia (Vittala et al., 2020). Using this technique, studies have demonstrated evidence of dysfunction in the frontal thalamocortical circuits in general and impaired cortical connectivity in the dorsolateral prefrontal cortex (DLPFC) in particular as compared to healthy controls using the single pulse paradigm (Li et al., 2021). Similarly, it is now possible to perform concurrent TMS/fMRI to interfere with specifically targeted networks and examining the cortical- and behavioral-level after-effects. fMRI exploits neurovascular coupling and can easily map TMS-evoked neuronal activity with high spatial resolution while providing a whole-brain coverage. However, owing to significant technical challenges, the concurrent TMS/fMRI setup currently exists in only a few specialized labs worldwide. The TMS coil has to be devoid of ferromagnetic material like other equipment in the MR environment. The TMS stimulator either has to be kept inside a shielded metal cabinet or outside the MR room, to which the coil then has to be connected using a waveguide. Additionally, a low-pass filter is necessary to filter out external high-frequency noise (Bungert et al., 2012a). Conventional MR radiofrequency (RF) coils pose obvious constraints on the positioning of the TMS coil. To surmount this issue, single-channel transmit/receive (Tx/Rx) volume ‘bird-cage’ coils can be used, which provide an adequate opening for optimal positioning of the TMS coil (Bestmann et al., 2003). However, these are single-channel RF coils which are insufficient for performing modern parallel multiband imaging sequences. Hence, some labs now use commercially available or custom-made flexible multichannel surface RF coil arrays (Wang et al., 2017; Oh et al., 2019) which can easily accommodate MR compatible TMS coils and also provide high-quality images for a concurrent TMS/fMRI study. To stabilize the TMS coil in a fixed position for the entire duration of the fMRI recording, customized MR compatible coil holders are required (Bestmann et al., 2003; Moisa et al., 2009).

The presence of a TMS coil between the subject’s head and the RF coil leads to local field inhomogeneities causing significant limitations in the signal-to-noise ratio (SNR). To overcome this, thinner MR compatible 7-channel surface RF-coil arrays have been developed, which can be mounted directly below the TMS coil. These novel arrays have been shown to achieve a five-fold rise in SNR at 3 cm depth underneath the TMS coil as compared to the bird-cage coils (Navarro de Lara et al., 2015, 2017). However, this arrangement can cause a reduction in the effective stimulation intensity and pose difficulties in reaching suprathreshold intensities in subjects with high motor threshold.

Use of concurrent TMS/fMRI can lead to static artifacts due to the presence of TMS coil on the magnetic field of the scanner, or cause dynamic artifacts during the actual discharging of the TMS coil. The field inhomogeneities caused by the TMS coil itself can be reduced using shimming techniques before image acquisition (Bungert et al., 2012b). Apart from these, eddy currents created by the changing MR fields in the copper windings of the TMS coil can be prevented using MR compatible TMS coils. Tiny leakage currents generated in the capacitors inside the TMS device can transmit through the coil, causing image artifacts (Weiskopf et al., 2009). These can be minimized using actively controlled high-voltage relay-diode systems to electrically insulate the TMS coil from the stimulator until immediately before and after each TMS pulse or by using built-in leakage filters (Weiskopf et al., 2009). Dynamic artifacts caused by the TMS pulse on the RF pulse can be prevented by setting precise time intervals between the two techniques (Bestmann et al., 2003; Navarro de Lara et al., 2017).

Clearly, setting up and running an adequately accurate system of acquiring concurrent TMS/fMRI data is contingent upon strong multidisciplinary technological expertise, timely quality control evaluations and a liberal financial support to acquire and maintain such state-of-the-art equipment. Once these aforementioned challenges have been met, TMS/fMRI can be used to effectively to make causal inferences based on a specific hypothesis. For example, based on the evidence of abnormal activity in the subgenual anterior cingulate cortex (sgACC) in individuals with depression, a study by Vink et al. (2018) assessed for the propagation of TMS-induced activity to sgACC after stimulation of left dorsolateral prefrontal cortex (DLPFC). Combined TMS/fMRI approaches facilitate a better understanding of brain physiology in general and psychiatric illnesses like schizophrenia in particular by overcoming the shortcomings of either technique alone. The following review provides an overview of the existing literature on multimodal TMS/fMRI in individuals with schizophrenia and potential future avenues.

## Materials and Methods

We conducted a systematic review was based on the recommended PRISMA guidelines^1^ using the PubMed electronic database. We searched for all publications whose titles or abstracts contained the following terms: magnetic resonance imaging OR functional MRI OR FMRI AND Transcranial magnetic stimulation OR TMS AND schizophrenia OR psychosis. We established the following inclusion criteria: (a) Experiments recruiting individuals with a diagnosis of schizophrenia, (b) use of single-/paired-pulse/repetitive TMS, and (c) use of resting-state or task-based fMRI. We included all kinds of publications such as case-control and open-label studies, case reports, and conference abstracts. Review articles and experiments which explored physiological processes in otherwise healthy subjects using TMS/fMRI were excluded. Both the authors conducted the searches and the selection process independently.

**FIGURE 1:**
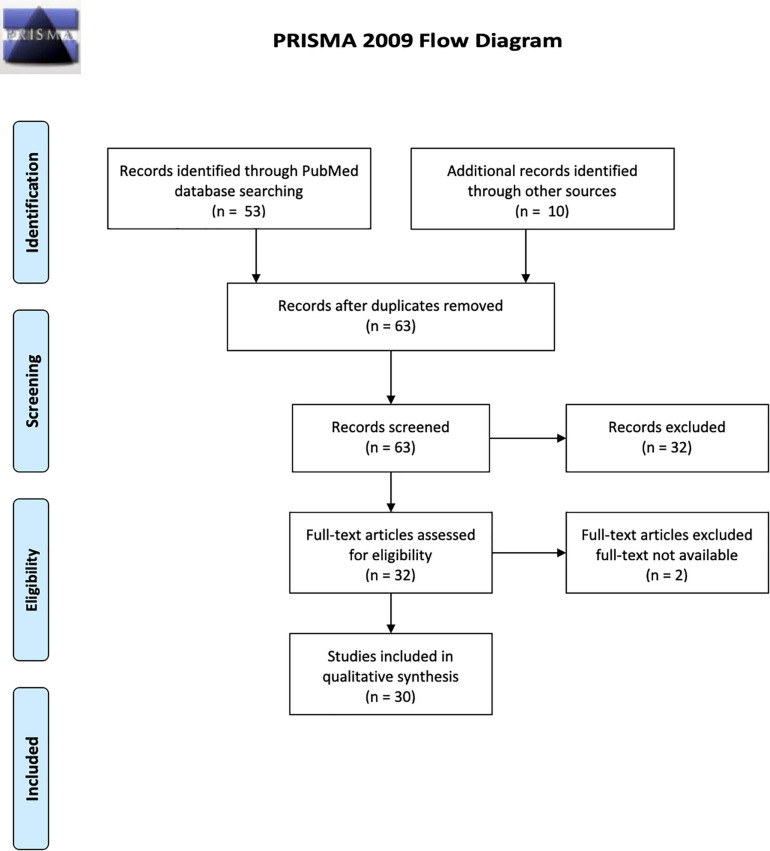
PRISMA diagram describing selection of studies for the review.

For the selected titles, full-text articles were retrieved, and reference lists of each were searched for additional publications. In case of incomplete or missing information, the corresponding author of the included studies were contacted. The initial search strategy yielded 53 results; after applying the selection criteria, 30 studies were included in the review based on both authors’ consensus. These were then categorized as those exploring schizophrenia pathophysiology (*n* = 6 studies) and those exploring treatment of schizophrenia (*n* = 24 studies). Figure 1 describes the flow diagram of the selection/inclusion process followed in this review.

## Results

### Multimodal TMS/fMRI Studies Exploring the Pathophysiology of Schizophrenia

A summary of all the 6 studies investigating the pathophysiology of schizophrenia has been presented in Table 1. These can be understood as those exploring cortical connectivity and those exploring cortical reactivity. Three of these experiments have utilized interleaved (concurrent) TMS/fMRI, while the rest have used it in a sequential or offline fashion by obtaining independent measurements and then correlating the two.

**TABLE 1:** Technical aspects and principal findings of TMS/fMRI studies exploring pathophysiology of schizophrenia.

Authors	Subjects	Concurrent pharmacotherapy	Investigation	What was being studied	How TMS and fMRI were combined	TMS target	Findings
Webler et al. (2020)	19 SZ 11 HC recruited. Final sample had 8 SZ and 11 HC	unmedicated	Cortical excitability and interhemispheric connectivity	L BA9 activation and FC between L and R BA9 compared to HC.	Concurrent TMS/fMRI	L DLPFC (BA9)	At equal TMS intensity, hyperexcitability in L BA9 and BA46 in SZ group HC showed ↑ activation in R BA9 implying better FC between L and R BA9.
Du et al. (2019)	24 SZ 30 HC	20 patients on antipsychotics, rest unmedicated. Those on BZDs excluded.	Middle Prefrontal-Motor Cortex connectivity	rsFC between M1 and PFC and its association with SICI	Motor cortex seed based whole brain rsfMRI and DTI done at baseline followed by ppTMS for measuring SICI	M1	↑rsFC between L PFC-M1 associated with ↑SICI and lesser symptoms ↓ FA at left CR in SZ group. SICI derived rsFC between L PFC-M1 had positive correlation with FA of left CR in SZ group.
Lindberg et al. (2016)	28 SZ or SZA 21 HS 31 HC	22 patients on antipsychotics, rest unmedicated. Those on AEDs, BZDs and antidepressants excluded.	Neural correlates of motor inhibition	SICI during a motor inhibition task (Stop Signal Task) and its relation to activity in Cortical inhibition network	TMS for obtaining SICI fMRI during Stop Signal Task	M1	↓ SICI during motor inhibition in SZ group despite equivalent motor inhibition performance as compared to HS and HC ↑ activation in B/l IFG, L MeFG during motor inhibition in SZ group compared to HC and ↑ activation in prefrontal, cingulate and pre-SMA compared to HS.
Guller et al. (2012a)	14 SZ 14 HC	All patients on antipsychotics	Aberrant thalamic functioning	Peak amplitude of thalamic response to cortical perturbation using spTMS	Concurrent spTMS/fMRI	L PCG	No difference in BOLD response of cortical tissue underlying site of stimulation ↓ response to spTMS in thalamus, mSFG and insula in SZ group. ↓ thalamus-mSFG and thalamus-insula effective FC in SZ group
Guller et al. (2012b)	14 SZ 14 HC	All patients on antipsychotics	Resting state functional connectivity, WM structural connectivity (FA) and GM integrity (VBM)	rsfMRI and structural (WM and GM) data using DTI	Concurrent spTMS/fMRI	L PCG	No rsFC differences between thalamus and PCG, thalamus and SFG, thalamus and insula, SFG and PCG, insula and PCG. No group differences in FA of tracts connecting spTMS-responsive voxels of thalamus and PCG, thalamus and SFG, thalamus and insula, PCG and SFG, PCG and insula ↓VBM measures in thalamus in SZ group compared to HC, but disappeared after correction for multiple comparisons. ↓VBM measures in R posterior insula in SZ group compared to HC. However, no difference in spTMS induced insular response between groups.
Hoffman et al., 2007	16 SZ divided into continuous hallucinators (*n* = 8) and intermittent hallucinators (*n* = 8)	All patients on psychotropics, details not provided.	Pathophysiology of AVH	Identifying cortical areas where TMS produces significant improvement in AVH	For intermittent AVH – BOLD maps of hallucination and non-hallucination periods were compared, while for continuous AVH, maps of BOLD signal correlations relative to functionally defined Wernicke’s area created to obtain 3–6 cortical sites; then probed using 1 Hz TMS in a crossover design. Clinical response correlated with fMRI findings.	Active – variable, based on AVH related activation patterns. Sham – TP area with coil angled 45^0^ off scalp.	In intermittent hallucinators, low levels of hallucination related activity in Broca’s area predicted greater L TPJ TMS rate of response. In continuous hallucinators, ↓ coupling between Wernicke’s area and right homologue of Broca’s area predicted greater L TPJ TMS rate of response.

*SZ = schizophrenia; SZA = schizoaffective disorder; HS = Healthy siblings; HC = healthy controls; a = active; s = sham; d = days; w = week; L = left; R = right; b/l = bilateral; spTMS = single pulse TMS; SICI = Short Interval Intracortical Inhibition; AVH = auditory verbal hallucinations; BA = Brodmann Area; DLPFC = Dorsolateral prefrontal cortex; PCG = precentral gyrus; SFG = Superior frontal gyrus; mSFG = medial superior frontal gyrus; IFG = inferior frontal gyrus; MeFG = medial frontal gyrus; M1 = Primary motor cortex; SMA = supplementary motor area; TP = temporoparietal; TPJ = temporoparietal junction; CR = corona radiata; ↑ = increases; ↓ = decreases; FC = functional connectivity; rsFC = resting state functional connectivity; rCBF = regional cerebral blood flow; BOLD = blood oxygen level dependent; DTI = diffusion tensor imaging; WM = white matter; GM = gray matter; FA = functional anisotropy; VBM = voxel based morphometry.*

#### Cortical Connectivity

Single-pulse TMS (spTMS) to the precentral gyrus has been utilized with concurrent fMRI to measure response in synaptically connected regions (thalamus, medial superior frontal cortex, insula) in a case-control study (Guller et al., 2012a). Schizophrenia patients showed reduced activation in the thalamus, medial superior frontal cortex, and insula response to spTMS to the precentral gyrus. Functional connectivity analyses revealed weaker thalamus-medial superior frontal cortex and thalamus-insula connectivity in patients, thereby demonstrating aberrant thalamic connectivity in schizophrenia (Guller et al., 2012a). In an extension of the experiment, resting state functional connectivity (rsFC), white matter (WM) structural connectivity, and gray matter (GM) integrity were assessed in the same subjects using DTI (Guller et al., 2012b). The study found impaired effective connectivity (measured using spTMS/fMRI) but normal functional connectivity (measured using resting state fMRI or rsfMRI) in schizophrenia patients and failed to find any WM or GM abnormalities that could explain the aberrant functional thalamic connectivity.

#### Cortical Reactivity

Short Interval Intracortical Inhibition (SICI) is a paired-pulse TMS paradigm that is known to be mediated by GABA_*A*_ receptors (Kujirai et al., 1993). Previous literature has consistently demonstrated SICI to be deficient in individuals with schizophrenia, implying a reduction in intracortical GABAergic neurotransmission (Radhu et al., 2013). A case-control study was conducted by Lindberg et al. to assess neural correlates of motor inhibition using concurrent fMRI/TMS. The study utilized a Stop Signal Task (SST) as a measure of volitional motor inhibition and the rapidity of inhibition process was estimated for each subject (labeled Stop Signal Reaction Time, SSRT). Simultaneously, motor evoked potentials (measure of cortical excitability) and SICI (measure of motor inhibition) were recorded during the stop-go task of the SST. Following this, fMRI data during motor inhibition was recorded using a modified version of the SST. The study demonstrated that despite having an equal motor inhibition performance on the SST, fMRI showed greater prefrontal and premotor activation in schizophrenia during the inhibition task than controls (Lindberg et al., 2016). This task-related modulation of SICI was notably higher in subjects who showed less inhibition-related activity in pre-SMA and cingulate motor area, providing direct evidence of task-related deficiency of SICI modulation. Another case-control study performed measurements of SICI, followed by seed-based whole-brain functional connectivity (FC) using the SICI stimulation site and diffusion tensor imaging (Du et al., 2019). Higher resting-state left prefrontal-motor cortex functional connectivity, accompanied by a higher functional anisotropy of left corona radiata was found to predict less inhibitory deficits (or higher SICI), implying that a top-down prefrontal influence might partly mediate the inhibitory deficits in the motor cortex in schizophrenia.

A recent case-control study by Webler et al. (2020) assessed for prefrontal excitability and interhemispheric functional connectivity using concurrent TMS/fMRI in schizophrenia patients and compared them with healthy controls. In both groups, resting motor threshold (RMT) was estimated at baseline and the left-sided DLPFC (Brodmann area 9) was then stimulated using 35 triplet TMS pulses at 100 ms apart (10Hz) at 0, 80, 100, and 120% of RMT in a randomized order. Simultaneously, fMRI was performed to assess for activation patterns in bilateral BA 9 and neighboring BA46. The study found that schizophrenia patients showed hyperexcitability in left-sided BA9 and BA46 compared to healthy controls for equal TMS intensity. Also, on stimulating the left BA9, healthy controls showed increased right-sided BA9 activity compared to schizophrenia patients, thereby demonstrating impaired interhemispheric connectivity in the patients (Webler et al., 2020).

#### Pathophysiology of Auditory Verbal Hallucinations

Apart from these studies, one study has investigated auditory verbal hallucinations (AVH) using TMS/fMRI (Hoffman et al., 2007). The study aimed to identify cortical sites where treatment with rTMS produced significant reduction in AVH and then assess statistical relationship between clinical response and fMRI changes in these regions. For this study, patients of schizophrenia with resistant AVH were divided into continuous or intermittent hallucinators. For intermittent hallucinators, BOLD activation maps comparing hallucination and non-hallucination periods were generated by using a behavioral task to demarcate onset and offset of each hallucination event. In continuous hallucinators, functionally defined Wernicke’s area was delineated in each case using the activation patterns generated while listening to external speech. Correlations between BOLD signal time course in Wernicke’s area, and other regions were used to map functional coupling to the former. In both groups, activation maps for AVH were then created around Wernicke’s area and 3–6 cortical sites for each case were identified. These were then probed using 1-Hz (16 min, once daily for 3 days) and sham rTMS using a crossover design. To the site producing greatest clinical benefit, 3 more days of active rTMS was administered after unmasking. The study demonstrated that temporoparietal areas of the dominant hemisphere were involved in experience of AVH and rTMS to these areas produced greater rates of improvement as compared to anterior temporal sites and sham stimulation. The study also demonstrated involvement of inferior frontal regions in the pathophysiology of AVH as suggested by higher levels of coactivation involving inferior frontal and temporoparietal areas during hallucination periods and a robust negative correlation between temporoparietal rTMS response and hallucination-related activation/coupling involving inferior frontal regions.

### Multimodal TMS/fMRI Studies Exploring the Treatment of Schizophrenia

A summary of all the studies exploring the treatment of schizophrenia has been presented in Supplementary Table 1. Additional details of the rTMS treatment-related parameters and outcome measures used in the studies have been provided in Table 2. Among positive symptoms of schizophrenia, the management of treatment-resistant AVH has been explored the most. These multimodal studies have either utilized fMRI for target localization (neuronavigation) or the comparison of pre-and post-treatment functional connectivity changes or both.

**TABLE 2:** TMS/fMRI studies on treatment of schizophrenia: rTMS parameters and outcome measures.

Authors	rTMS parameters	Number of sessions	Outcome measure/assessment
**Hallucinations**			
Slotema et al. (2011)	1 Hz, 90% RMT, 15 min	15 (3w)	AHRS, Positive subscale of PANSS, PSYRATS at baseline, weekly for 3w and monthly follow-up for 3m
Paillère-Martinot et al. (2017)	1 Hz, 100% RMT, 20 min	10	SAPS, AHRS at baseline and last day of treatment.
Vercammen et al. (2010)	1 Hz, 90% RMT, 20 min	12 (twice daily)	P3 item of PANSS before and after treatment. Brain activity in B/L TPJ, IFG, ACC, amygdala and insula.
Bais et al. (2017)	1 Hz, 90% RMT, 20 min (for B/l group, 10 min on each side)	12 (twice daily)	P3 item of PANSS and AHRS before and after treatment. Effect of treatment on network connectivity within and between components of DMN, ASM, SAL, LFP, RFP and BFT during a word evaluation task.
de Weijer et al. (2014)	1 Hz, 90% RMT, 20 min and 20 Hz, 80% RMT, 13 trains, 10 s on, 50 s off	Daily for 5 days, then weekly maintenance for 3w (total 8 sessions)	AHRS at baseline, after 5d and after 3w of maintenance treatment.
Schönfeldt-Lecuona et al. (2004)	1 Hz, 90% RMT, 16 min	5	Haddock self-rating scale at baseline and after treatment
Kindler et al. (2013)	1Hz Group (*n* = 8): 1 Hz, 90% RMT, Day 1: 8 min Day 2: 12 min Day 3–10: 16 min TBS Group (*n* = 7): cTBS 30Hz Day 1–3:4 × 801 pulses (total 3,204 pulses); Day 4–10:2 × 801 pulses (total 1,602 pulses)	10	PANSS, PSYRATS at baseline and after treatment
Maïza et al. (2013)	20 Hz, 80% RMT, 13 trains, 10s on, 50s off (only to SZ group)	4 (twice daily)	AHRS Correlation between L pSTS activity and AHRS Correlation between mean GM volume and activation in L pSTS
Briend et al. (2017)	20 Hz, 80% RMT, 13 trains, 10 s on, 50 s off (only SZ group)	4 (twice daily)	AHRS at baseline and d12 Comparison of baseline FC in L pSTS between SZ and HC Correlation between FC and AHRS
Fitzgerald et al. (2007)	1 Hz, 90% RMT, 15 min	10	PANSS, auditory hallucinations subscale of PSYRATS, HCS weekly.
Homan et al. (2012)	1Hz Group: 1 Hz, 90% RMT Day 1: 8 min Day 2: 12 min Day 3–10: 16 min TBS Group: cTBS 30Hz Day 1–3:4 × 801 pulses (total 3,204 pulses); Day 4–10:2 × 801 pulses (total 1,602 pulses)	10	Comparison of resting rCBF in L STG between responder (AHRS reduction ≥ 50%) and non-responders
Sommer et al. (2007)	1 Hz, 90% RMT, 20 min	15 (3w)	AHRS, positive scale of PANSS at baseline, end of each treatment week and follow-up at 6 and 13w from baseline
Montagne-Larmurier et al. (2009)	20 Hz, 80% RMT, 13 trains, 10 s on, 50 s off	4 (twice daily)	AHRS at baseline and d12
Zöllner et al. (2020)	cTBS 30Hz Day 1–3:4 × 801 pulses (total 3,204 pulses); Day 4–10:2 × 801 pulses (total 1,602 pulses)	10	Comparison of brain activation (PAC) at baseline vs. remission of AVH using an auditory stimulation paradigm
Giesel et al. (2012)	1 Hz Week 1: 80% RMT, 10 min Week 2: 100% RMT, 10 min Week 3: 100% RMT, 20 min Week 4: 100% RMT, 20 min along with external verbal stimulation during ITI.	20 (4w)	AHRS at baseline and weekly. Brain activity during AVH and during external verbal stimulation.
Jardri et al. (2008)	1 Hz, 100% RMT, 1000 pulses/session	10	VAS, SF-36 at baseline and after treatment
Jardri et al. (2007)	1 Hz	10 (sessions repeated every 5w)	AHRS, CGAS at baseline and after treatment.
**Negative symptoms**			
Brady et al. (2019)	iTBS 50Hz, 100% AMT, 2s on, 8s off, total 600 pulses	10 (twice per day)	Baseline rsfMRI and SANS in network discovery cohort. rsfMRI and PANSS at baseline and after treatment (in network validation cohort)
Basavaraju et al. (2019)	iTBS 50Hz, 80% AMT, 2s on, 8s off, total 600 pulses	10 (twice per day)	Seed based rsfMRI and SANS at baseline and after treatment.
Dlabac-de Lange et al. (2015)	10 Hz, 90% RMT, 20 trains, 10s on, 50s off	30 (twice per day)	SANS, PANSS Negative subscale at baseline and after treatment. Performance in ToL task i/f/o reaction time and accuracy pre- and post-treatment. Effect of treatment on brain activation during ToL task
**Neurocognition**			
Prikryl et al. (2012)	10 Hz, 110% RMT, 15 trains, 10s on, 30s off	15 (3w)	PANSS and neuronal activation during VFT task at baseline and after treatment.
Guse et al. (2013)	10 Hz, 110% RMT, 10 trains, 10s on, 30s off	15 (3w)	Activation patterns during letter 2-back task at baseline and after treatment
**Social cognition**			
Liemburg et al. (2018)	10 Hz, 90% RMT, 20 trains, 10s on, 50s off	15 (3w)	Activation patterns during Wall of Faces (social-emotional evaluation) task before and after treatment.
**Agency**			
Jardri et al. (2009)	1 Hz, 100% RMT, total 1000 pulses	10	Self-other discrimination tasks (Motor agency, source monitoring and speech awareness) and activation patterns in agency network during them. AHRS, CGAS

*S = second; d = days; w = week; m = month; L = left; R = right; b/l = bilateral; RMT = resting motor threshold; AVH = auditory verbal hallucinations; TP3 – midpoint of the line joining T3 to P3 as per EEG 10–20 system; TP4 – midpoint of the line joining T4 to P4 as per EEG 10–20 system; TPJ – temporoparietal junction; TPC – temporoparietal cortex; STG = superior temporal gyrus; MTG = middle temporal gyrus; AG = angular gyrus; HG = Heschl’s gyrus; SMG = supramarginal gyrus; PAC = primary auditory cortex; Spt = Sylvian parietotemporal; pSTS = posterior superior temporal sulcus; SSC = somatosensory cortex; PFC = prefrontal cortex; DLPFC = dorsolateral prefrontal cortex; ACC = anterior cingulate cortex; PCC = posterior cingulate cortex; MFG = middle frontal gyrus; IFG = inferior frontal gyrus; MeFG = medial frontal gyrus; FP = frontoparietal; IPL = inferior parietal lobule; DMN = default mode network; ASM = auditory sensorimotor network; SAN = salience network; LFP = left frontoparietal network; RFP = right frontoparietal network; BFT = bilateral frontotemporal network; ↑ = increases; ↓ = decreases; AHRS = Auditory Hallucinations Rating Scale; PANSS = Positive And Negative Syndromes Scale; PSYRATS = Psychotic Symptom Rating Scales; SAPS = Scale For The Assessment of Positive Symptoms; SANS = Scale For The Assessment of Negative Symptoms; HCS = Hallucination Change Score; VAS = Visual Analog Scale; SF36 = 36 item Short Form survey; CGAS = Childrens Global Assessment Scale; ToL = Tower of London; VFT = verbal fluency task; ASL = arterial spin labeling; GM = gray matter; FC = functional connectivity; rCBF = regional cerebral blood flow.*

#### Hallucinations

Most studies have utilized block design fMRI for target localization using language tasks to create individualized cortical targets of auditory processing areas for treatment using rTMS. This is based on the hypothesis that abnormalities in the speech/language network underlie the pathophysiology of AVH in schizophrenia (Hoffman et al., 1999; Gavrilescu et al., 2010; Oertel-Knöchel et al., 2014). Others have utilized the event-related/symptom capture fMRI paradigm to create individualized activation maps for target localization (Sommer et al., 2007; Slotema et al., 2011; de Weijer et al., 2014). The second group of multimodal studies has utilized fMRI to assess whether rTMS leads to functional connectivity changes in the areas implicated in AVH and whether these changes correlate with clinical improvement. Based on the existing literature, the efficacy of fMRI-guided rTMS over sham for AVH has not clearly been established (Schönfeldt-Lecuona et al., 2004; Slotema et al., 2011; de Weijer et al., 2014; Paillère-Martinot et al., 2017).

Similarly, studies directly comparing fMRI guided and non-guided (10/20 EEG system based) treatment of AVH has also not found any superiority of the former over the latter (Sommer et al., 2007; Slotema et al., 2011). However, there has been evidence from some sham-controlled studies that active rTMS to the temporoparietal junction affects local speech-related network as well as its connections to distal networks (Vercammen et al., 2010; Bais et al., 2017). The first study demonstrated increased connectivity between left temporoparietal junction (TPJ) and right insula secondary to active treatment (Vercammen et al., 2010). The second study compared the effects of left TPJ, bilateral TPJ, and sham stimulation on inner speech-related brain networks (Bais et al., 2017). It showed that active rTMS to the left or bilateral TPJ areas resulted in a weaker network contribution of the left supramarginal gyrus to the bilateral frontotemporal network, which was hypothesized to a reduced likelihood of speech intrusions. However, only left TPJ stimulation led to stronger network contributions of right superior temporal gyrus to functional areas involved in attention and cognitive control, hinting toward the possible superiority of left TPJ stimulation to bilateral TPJ stimulation.

Apart from AVH, management of treatment resistant coenesthetic hallucinations has also been explored in a single case study. Based on activity in bilateral somatosensory cortices (SSC) during active hallucinations using data-driven analyses, the patient was administered 10 days of neuronavigated 1Hz rTMS over SSC, with which the frequency and intensity of coenesthetic hallucinations decreased (Jardri et al., 2008). However, sham stimulation of the same site was not tried prior to the active stimulation.

#### Negative Symptoms

Only three studies have specifically examined efficacy of rTMS for treating negative symptoms using multimodal TMS/fMRI approach (Dlabac-de Lange et al., 2015; Basavaraju et al., 2019; Brady et al., 2019). The earliest of these, a double-blind randomized sham controlled trial (RCT), examined the effect of 3 weeks of 10 Hz rTMS to bilateral DLPFC (located using EEG 10–20 system) on frontal brain activation in patients with negative symptoms of schizophrenia, as measured by fMRI during the Tower of London (ToL) task (Dlabac-de Lange et al., 2015). The study demonstrated an increased activity in the right DLPFC and right medial frontal gyrus in the active arm, which was accompanied by significant improvement in negative symptoms as compared to the sham arm. The second RCT employed a different approach by using rsfMRI to identify functional connectivity correlates of negative symptoms (Brady et al., 2019). The study found the functional connectivity breakdown between the right DLPFC and the midline cerebellar node in the default network as the most significant predictor of negative symptom severity in a network discovery cohort. Five days of twice daily cerebellar intermittent theta burst stimulation (iTBS) led to improvement in negative symptoms and this was associated with the reversal of functional dysconnectivity in an independent cohort. However, a subsequent sham controlled RCT of 5 days of twice daily iTBS to cerebellar vermis demonstrated a significant but equal improvement in negative symptoms in both active and sham groups at the end of treatment and at 6-week follow-up (Basavaraju et al., 2019). Nevertheless, only the active TMS group showed a significant engagement of the cerebellar-prefrontal resting-state functional connectivity.

#### Cognitive Symptoms

Two studies have examined the effect of rTMS on cognition in schizophrenia using task-based fMRI (Prikryl et al., 2012; Guse et al., 2013). Both of these have assessed for improvement in performance of working memory (WM) tasks (verbal fluency and letter 2-back) along with changes in neuronal activation during task-based fMRI using a double-blind sham-controlled design. The first trial found equal improvement in WM task performance in both arms and failed to show any differences in task-based activation in either groups (Prikryl et al., 2012). The second trial also utilized an additional healthy control arm to compare baseline and post-treatment scores with schizophrenia patients. The study did not find any differences in WM task-based activation between schizophrenia patients and healthy controls after 3 weeks of 10Hz rTMS (110% RMT, ITI 30 s, 1000 stimuli per session) or surprisingly, even at baseline (Guse et al., 2013).

#### Social Cognition

Only one sham controlled study has indirectly assessed the role of rTMS in social cognition in schizophrenia using multimodal approach (Liemburg et al., 2018). The RCT primarily assessed for activity changes in the prefrontal cortex during an ambiguous socio-emotional processing (Wall of Faces) task at baseline and compared with those after 3 weeks of 10Hz rTMS to bilateral DLPFC. It demonstrated a reduction in task-based activation in frontal, parietal and striatal regions, which they hypothesized to be possibly secondary to more effective processing in the prefrontal brain networks secondary to active treatment.

#### Agency

The role of rTMS in self-agency has been examined in a case study of childhood-onset schizophrenia who had persistent self-awareness impairments along with resistant AVH (Jardri et al., 2009). Based on abnormal activation in the right inferior parietal lobule (IPL) and related self-awareness network during self-agency related tasks (collision paradigm for motor-agency, block design experiment for speech awareness, and two scales for source monitoring), the patient was administered 10 days of 1Hz rTMS to the right TPJ. There was an improvement in the performance of self-other discrimination tasks associated with increased activity in the right IPL. However, there was no improvement in AVH until the patient was also administered a course of 1Hz rTMS to left TPJ, suggesting a functional dissociation between self-agency and hallucinations related networks.

## Discussion

The studies reviewed here illustrate the variety of concurrent TMS/fMRI experiments that have been conducted in patients with schizophrenia. These include isolated case reports, open-label and randomized control trials. The studies exploring treatment have assessed for effects of rTMS on hallucinations, negative symptoms, neurocognition, social cognition, and agency, while the studies exploring pathophysiology have in general looked at altered cortical excitability or connectivity in schizophrenia as compared to healthy controls.

### Studies Exploring Pathophysiology

The findings of multimodal studies evaluating cortical reactivity are different from that of prior research. While the study by Lindberg et al. (2016) did demonstrate a task-related deficiency in SICI during motor inhibition, this was associated with increased motor inhibition-related processing in the prefrontal and premotor areas. Previous studies have, in general, shown decreased prefrontal activation response inhibition tasks in schizophrenia patients (Kaladjian et al., 2007; Hughes et al., 2012).

The experiments on cortical connectivity demonstrated impaired effective connectivity between the thalamus and insula and thalamus and superior frontal gyrus, thereby implicating thalamic abnormalities in the pathogenesis of schizophrenia (Guller et al., 2012a,b). This is in line with previous neuropathological and neuroimaging research that has demonstrated thalamic dysfunction in schizophrenia (Clinton and Meador-Woodruff, 2004; Harms et al., 2007). An important point worth mentioning here is that while functional connectivity primarily provides an index of coactivation of two or more brain regions, it does not give any information as to the causal or primary contribution of one area over the other. In contrast, TMS-fMRI can help to infer causal influences of one brain region over the other via effective connectivity (Friston, 2011). This could help understand the heterogeneity from rs-fMRI studies by creating a better characterization of intra and inter-individual variability, thus paving the way for a more tailor-made or personalized approach toward treatment using rTMS. Findings of altered prefrontal interhemispheric connectivity in the study by Weber et al. (Webler et al., 2020) parallel those in previous TMS studies on motor cortex, which have demonstrated transcallosal inhibition abnormalities in patients of schizophrenia (Boroojerdi et al., 1999; Fitzgerald et al., 2002). Previous structural neuroimaging studies have also pointed toward corpus callosum impairments in schizophrenia (Foong et al., 2000; Keshavan et al., 2002).

Left TPJ is probably the commonest targeted area in treating persistent AVH using various non-invasive brain stimulation modalities. While other areas such as the inferior frontal gyrus (IFG; Broca’s area or its right homologous region) might be considered as potential targets for treating AVH based on the activation patterns during AVH, the exploratory study by Hoffman et al. (2007) demonstrated no improvement in delivering rTMS to these areas and also underscored the importance of left-sided TPJ stimulation for treatment of AVH.

An important point worth mentioning here is that a variety of psychotropics, including antidepressants, antipsychotics, mood stabilizers and benzodiazepines can have an effect on TMS measures of cortical excitability (Ziemann et al., 2015). Antiepileptic mood stabilizers are known cause an increase in values in RMT while BZDs are known to increase SICI. Antipsychotics such as haloperidol have also been noted to decrease SICI. Similarly, concurrent administration of psychotropics can also have effects on TMS measures of cortical plasticity. Antipsychotics like Haloperidol and Sulpiride have been shown to suppress plasticity induced by various NIBS methods. Mood stabilizers such as lamotrigine can reduce LTP-like plasticity. Whereas, SSRIs such as citalopram have also been shown to promote LTP-like plasticity and abolish LTD-like plasticity. These effects have in general been shown to persist and normalize only after withdrawal of the drug. While conducting studies that explore pathophysiology of schizophrenia, there are obvious difficulties in recruiting patients who are unmedicated and in acute phase of psychosis. It is not surprising that subjects in all studies in the current review barring one (Webler et al., 2020) were on antipsychotics at the time of assessment. Some of these studies excluded those on mood stabilizers and benzodiazepines at the time of assessment, while others had subjects who were on stable doses of benzodiazepines, mood stabilizers and antidepressants at the time of assessment. When compared to health controls who are essentially drug free, it is expected that the findings related to cortical excitability and plasticity will be altered to an extent by concurrent administration of psychotropics.

### Studies Exploring Treatment

Functional magnetic resonance imaging-based target localization for rTMS offers a promising approach toward providing personalized therapy for various symptom domains of schizophrenia. However, current research has not proved unequivocally whether this approach is superior to the conventional 10/20 EEG based system. Most studies have been conducted on patients with medication-resistant symptoms and are plagued by methodological concerns stemming from small sample sizes and shorter duration of treatment/number of pulses. Moreover, the cost-effectiveness of such a treatment in clinical settings in terms of time, money, and workforce also needs to be considered.

There are also particular concerns with regards to fMRI-based neuronavigated rTMS, which merit a mention. Target localization for AVH in current multimodal studies has been performed using event related fMRI or block design fMRI, both of which are types of task-based fMRI. The main caveat with task-based fMRI is that the demonstration of functional connectivity between two regions does not imply a causal relationship or even a direct connection between the said regions. Furthermore, block design paradigm of fMRI for AVH is based on the concept that areas related to language processing are the same ones involved in the pathogenesis of AVH, which need not be necessary. Similarly, targets determined using both block-based and event-related fMRI can include structures that are deeper and even inaccessible to TMS. Target localization for negative symptoms has been performed using seed based rsfMRI, which at best can provide only indirect measurements of neural activity. In comparison to this, using concurrent TMS/fMRI might help in a more efficient localization of target by allowing to observe immediate response to local perturbation and also provide direct proof of target engagement (Windischberger, 2019; Bergmann et al., 2021).

Furthermore, considering that psychiatric illnesses involve abnormalities in complex neural networks, it seems too simplistic and reductionistic to expect that stimulation or inhibition of a single area will improve symptoms. Recent research in obsessive-compulsive disorder and depression has shown that a deeper and broader area of stimulation targeting subcortical regions using deep TMS may be a better alternative to the focal cortical stimulation using the F8 coil (Tendler et al., 2016; Lusicic et al., 2018). It has been suggested that deep-TMS might be more helpful due to targeting more widespread networks, thus questioning the need for functional imaging (Tendler et al., 2016).

The utility of rTMS is limited by its depth of penetration, making it possible to target only superficial cortical structures. However, combining fMRI with TMS also enables us to examine the effects of stimulating superficial cortical structures on deeper connections. This has been utilized in studies in healthy subjects to understand various aspects of brain physiology. For example, Zito et al. (2020) found the network-related sense of agency in healthy subjects to be amenable to inhibition by low-frequency rTMS. Another study by Hermiller et al. (2020) attempted theta burst stimulation to the hippocampal network targeted location in the parietal cortex during concurrent fMRI while performing a memory task and demonstrated increased activity of the targeted hippocampus during scene encoding and subsequently increased recollection. Such insights obtained from studies in healthy subjects could help understand physiological mechanisms and plan future experiments in patients with schizophrenia.

The current review has certain limitations. The main objective of this review was to discuss concurrent TMS/fMRI studies in schizophrenia. Hence, studies in normal healthy individuals that have investigated physiological mechanisms that might be aberrant in schizophrenia (for example, potential pathways for AVH, sense of agency) were excluded from this study. Also, this review has only examined studies published in the English language and may have missed out studies published in non-English languages. Most studies that have been reviewed here have not utilized interleaved TMS/fMRI, possibly due to the aforementioned technical and methodological challenges. Future studies using TMS/fMRI will require further optimization of these challenges while also using proper sham conditions to improve the quality of the studies. Correspondingly, further technical refinements in the entire process of concurrent TMS/fMRI are necessary so that these can be easily replicated across different centers. In conclusion, there is a definitive role of experiments combining TMS and fMRI in schizophrenia. Larger and adequately powered multicentric trials employing combined TMS/fMRI are needed to get consistent and reliable results. Such multimodal techniques appear to be a promising approach in elucidating the pathophysiology of schizophrenia and could also open up a possibility toward the development of a personalized approach toward treatment of its debilitating symptoms.

## Author Contributions

SB and UM performed the literature review. SB prepared the first draft. UM supervised and SB edited the manuscript. Both authors contributed to the article and approved the submitted version.

## Conflict of Interest

The authors declare that the research was conducted in the absence of any commercial or financial relationships that could be construed as a potential conflict of interest.

## Publisher’s Note

All claims expressed in this article are solely those of the authors and do not necessarily represent those of their affiliated organizations, or those of the publisher, the editors and the reviewers. Any product that may be evaluated in this article, or claim that may be made by its manufacturer, is not guaranteed or endorsed by the publisher.

## Footnotes

1
http://www.prisma-statement.org/
