# Supplementary material for: A Review of Studies Leveraging Multimodal TMS-fMRI Applications in the Pathophysiology and Treatment of Schizophrenia
Source: Front Hum Neurosci. 2021 Aug 2;15:662976. doi: 10.3389/fnhum.2021.662976 (PMC8372850; doi:10.3389/fnhum.2021.662976)
Supplement: Supplementary file 1 [file Table_1.docx]

**Table S1: Summary and principal findings of TMS/ fMRI studies exploring treatment of schizophrenia**

| **Author** | **Type of study** | **Subjects** | **Concurrent pharmacotherapy** | **Role of fMRI** | **rTMS target** | **Findings** |
| --- | --- | --- | --- | --- | --- | --- |
| **Hallucinations** |  |  |  |  |  |  |
| Slotema et al. (2011)(1) | Randomised, double-blinded, placebo controlled | 20 SZ fMRI guided, 22 SZ left TP,  20 SZ sham  (Medication resistant AVH) | FGAs, SGAs, Lithium and antidepressants, doses not known. Those on AED and BZD excluded. | Target localisation | fMRI group and sham group: variable, based on individual AVH related activation patterns  Left TP group: TP3 | ↓ in AHRS scores across all 3 groups; no group differences.  No differences in PANSS Positive subscale and PSYRATS across the 3 groups. |
| Paillère-Martinot et al. (2016)(2) | Randomised, double blinded, placebo controlled | 15 SZ active  12 SZ sham  (medication resistant AVH) | Active: 474.40 mg (± 84.33)  Sham: 547.92 mg (± 94.29) in  CPZE | Target localisation | STG in 14 and MTG in 13 patients based on a language recognition task | Sig ↓ in SAPS hallucination subscale in both arms; no difference between groups. Those with external AVH improved more than those with internal AVH. |
| Vercammen et al. (2010)(3) | Randomised, double-blinded, placebo controlled | 9 SZ active  9 SZ sham  (medication resistant AVH) | On stable dose, details not known | fMRI change | TP3 | Trend level significant improvement in AVH with a rTMS, but no changes in connectivity between TPJ, ACC and amygdala.  a rTMS ↑ connectivity between L TPJ and R insula |
| Bais et al. (2017)(4) | Randomised, double blinded, placebo controlled | Left rTMS (n=7)  B/l rTMS (n=9)  Sham (n=8)  (medication resistant AVH) | FGAs and SGAs; doses not known. | fMRI change | TP3, TP4 | PANSS P3 – trend towards decreased score in left rTMS group, no change in B/l and sham groups; no group differences in AHRS total change scores.  Left rTMS caused ↓ network contribution of L SMG to BFT and  ↑ network contribution of R STG to ASM, R IFG to LFP, L MFG to DMN. |
| De Weijer et al. (2014)(5) | Randomised, double-blinded | 18 SZ randomised to 1Hz (n=10) and 20Hz group (n=8)  (medication resistant AVH) | 1 Hz group: 733 mg (± 417) in CPZE  20 Hz group: 545 mg (± 379) in CPZE. Those on AED and BZD excluded. | Target localisation | Cluster with highest signal change in the area containing b/l AG, HG and SMG based on individual AVH related activation patterns | ↓ in AHRS scores in both groups after 5d. No difference between the groups.  No treatment effect after 3w of follow-up treatment. |
| Schönfeldt-Lecuona et al. (2004)(6) | Cross-over sham controlled | 12 SZ  (medication resistant AVH) | 616.66 mg (± 461.57) in CPZE. BZD stopped 1 week before beginning of TMS. | Target localisation  (all underwent structural imaging, fMRI in additional 6) | PAC or Broca’s area using block design. Midline parieto-occipital in sham. Stimulated in  randomised order. | No significant reduction in hallucination severity |
| Kindler et al. (2013)(7) | Open-label | 15 SZ rTMS  vs 15 SZ TAU  (medication resistant AVH) | rTMS group: 591.1 mg (± 254.1)  TAU group:  479.9 mg (± 241.0) in CPZE | Target localisation and fMRI change | Area Spt based on a language task | ↓ rCBF in PAC, L Broca and cingulate gyrus in rTMS group.  No differences in rCBF decrease between 1Hz and TBS groups  ↓ rCBF in PAC correlated with ↓ in AVH scores. |
| Maïza at al. (2013)(8) | Open-label | 9 SZ  9 HC  (medication resistant AVH) | 626 mg (± 598) in CPZE | Target localisation and fMRI change | L pSTS, based on a language task | Pre-treatment activity in L pSTS negatively correlated with AHRS.  Post-treatment ↓AHRS, no change in L pSTS activity, along with decoupling of correlation.  Positive correlation between mean GM volume and activation in L pSTS. |
| Briend et al. (2017)(9) | Open-label | 11 SZ  10 HC | 575 mg (± 548) in CPZE. In 1 patient, dose of APD ↑ during treatment; 8 patients also on stable dose of BZD. | Target localisation and fMRI change | L pSTS, based on a language task | Reduced FC in L pSTS region in SZ as compared to HC at baseline  Sig ↓ in AHRS scores after treatment  No correlation between AHRS and FC before and after treatment |
| Fitzgerald et al. (2007)(10) | Open label | 3 SZ, 4 HC (medication resistant AVH) | 750 mg (± 278.38) in CPZE. One patient additionally on Na Valproate 1500md/d and; another on Diazepam 15mg/d | fMRI change | TP3 | ↑ task related activation in language processing areas including L TPC |
| Homan et al. (2012)(11) | Open-label, rater-blinded | 24 SZ or SZA  (medication resistant AVH) divided into 1Hz (n=12) and cTBS (n=12) group | Responders: 448.9 mg (± 132.2)  Non-responders:  586.2 mg (± 257.1) in CPZE | Target localisation and fMRI change using ASL. | Area Spt in L STG based on a language task | Responders (AHRS reduction ≥50%, n=9) had higher resting rCBF in L STG as compared to non-responders. |
| Sommer et al. (2007)(12) | Open label | 7 SZ fMRI guided,  6 SZ non-guided  (medication resistant AVH) | No details | Target localisation | Based on individual AVH related activation patterns or TP3 if no activation map acquired. | ↓ severity of AVH in both groups, persisting at 13w from baseline; no group differences.  Severity of psychosis showed trend towards more improvement in fMRI guided group. |
| Montagne-Larmurier et al. (2009)(13) | Open label | 11 SZ  (medication resistant AVH) | 575 mg (± 548) in CPZE. In 1 patient, dose of APD ↑ during treatment; 8 patients also on stable dose of BZD. | Target localisation | L pSTS based on a language task | ↓ global severity and frequency of AVH. 2 patients reported no AVH at 6 months. |
| Zöllner et al. (2020)(14) | Case study | 1 VLOSLP, treated with TBS during 2 independent episodes | 1^st^ episode: Olanzapine 20mg/d  2^nd^ episode:  Olanzapine 15mg/d | fMRI change | TP3 | ↓ activation of L PAC during remission of AVH |
| Giesel et al. (2012)(15) | Case study | 1 SZ  (medication resistant AVH) | Clozapine 550mg/d, Amisulpride 400mg/d, Lithium 800mg/d | fMRI change | 2cm above T3 (L STG) | ↓ frequency of AVH.  Activation of insula and operculum remained stable.  ↑ activation in the temporal cortex during external verbal stimulation. |
| Jardri et al. (2008)(16) | Case study | 1 SZ  (medication resistant) | No details | Target localisation | SSC based on activity during coenesthetic hallucinations. | 55% reduction in hallucinations; frequency: 73% to 18%; intensity: 84% to 30%, maintained for 8w |
| Jardri et al. (2007)(17) | Case study | 1 COS  (medication resistant AVH) | No details | Target localisation | PAC based on AVH related activation. | AVH completely stopped. 40% improvement in CGAS. |
| **Negative symptoms** |  |  |  |  |  |  |
| Brady Jr et al. (2019)(18) | Randomised, double-blinded, placebo controlled | 35 SZ and 9 SZA for network discovery  11 SZ for clinical trial | Network discovery cohort: 305.3 mg (± 232.6)  Clinical trial: 614.2 mg (± 606.5) in CPZE | Target localisation (in network discovery cohort) and fMRI change (in network validation cohort) | Midline cerebellar vermis | FC between R DLPFC and midline cerebellar node predicted negative symptom severity  a rTMS more effective in ↓ negative symptom severity and ↑ DLPFC-cerebellar FC |
| Basavaraju et al. (2019)(19) | Randomised, double-blinded, placebo controlled | 30 SZ active  30 SZ sham | No details | fMRI change | Midline cerebellar vermis | ↓ SANS scores in both arms, no group differences.  However, ↑ R PFC-cerebellar FC with a rTMS. |
| Dlabac-deLange et al. (2015)(20) | Randomised, double-blinded, placebo controlled | 24 SZ with PANSS Negative subscale score ≥15 randomized to active (n=11) and sham (n=13) | Clozapine, Olanzapine, Risperidone, Paliperidone, Aripiprazole, Haloperidol and other typicals. No details of doses. | fMRI change | b/l DLPFC (F3 and F4) | a rTMS caused ↓ SANS scores compared to sham, upto 3m followup.  Active group showed ↑ activity in R DLPFC, R MeFG and ↓ activity in L PCC after treatment.  No differences in cognitive measures post-treatment between the groups.  No correlation between improvement and changes in brain activation. |
| **Neurocognition** |  |  |  |  |  |  |
| Prikryl et al. (2012)(21) | Randomised, double-blinded, placebo controlled | 30 SZ randomized to active (n=19) and sham (n=11) | Active: 328.95 mg (± 143.44)  Sham: 304.55 mg (± 140.01) in  CPZE | fMRI change | L DLPFC | ↓ negative symptoms in both groups, active > sham  Equal ↑ in mean VFT score in both groups  No differences in neuronal activation during VFT task after rTMS treatment in either groups. |
| Guse et al. (2013)(22) | Randomised, double-blinded, placebo controlled | 25 SZ  22 HC  Both groups randomised to active and sham | Stable dose of SGAs, details not known. AEDs and high dose BZSs avoided. | fMRI change | F3 | Both SZ and HC showed equal activity in FP and subcortical regions during WM task  No activity change in these regions overtime in either groups. |
| **Social cognition** |  |  |  |  |  |  |
| Liemburg et al. (2018)(23) | Randomised, double-blinded, placebo controlled | 22 SZ with PANSS Negative subscale score ≥15 randomised into 11 active and 11 sham | Active: 7.6 mg (± 3.5)  Sham: 8.7 mg (± 8.0) in  Haloperidol equivalents | fMRI change | B/l DLPFC (F3 and F4) | ↓ activation of frontal, parietal and striatal regions during Wall of Faces (social-emotional evaluation) task after a rTMS; whereas,  ↑ activation compared to baseline after s rTMS |
| **Agency** |  |  |  |  |  |  |
| Jardri et al. (2009)(24) | Case study | 1 COS | Hydorxyzine, Lorazepam up to 10mg/d | Target localisation and fMRI change | 1^st^ treatment: R TPJ (for self-agency),  L TPJ (for AVH)  2^nd^ treatment: L TPJ for both | 1^st^ treatment: Improved scores on self-other discrimination tasks associated with ↑ activity in R IPL after treatment to R TPJ. AVH improved only after L TPJ targeted.  2^nd^ treatment: both symptoms improved with L TPJ rTMS |

SZ = schizophrenia; SZA = schizoaffective disorder; COS = childhood onset schizophrenia; VLOSLP = very late onset schizophrenia like psychosis; HC = healthy controls; a = active; s =sham; TAU = treatment as usual; d = days; w = week; L = left; R = right; b/l = bilateral; AVH = auditory verbal hallucinations; TP3 – midpoint of the line joining T3 to P3 as per EEG 10-20 system; TP4 – midpoint of the line joining T4 to P4 as per EEG 10-20 system; TPJ – temporoparietal junction; TPC – temporoparietal cortex; STG = superior temporal gyrus; MTG = middle temporal gyrus; AG = angular gyrus; HG = Heschl’s gyrus; SMG = supramarginal gyrus; PAC = primary auditory cortex; Spt = Sylvian parietotemporal; pSTS = posterior superior temporal sulcus; SSC = somatosensory cortex; PFC = prefrontal cortex; DLPFC=dorsolateral prefrontal cortex; ACC = anterior cingulate cortex; PCC = posterior cingulate cortex; MFG = middle frontal gyrus; IFG = inferior frontal gyrus; MeFG = medial frontal gyrus; FP = frontoparietal; IPL = inferior parietal lobule; DMN = default mode network; ASM = auditory sensorimotor network; SAN = salience network; LFP = left frontoparietal network; RFP = right frontoparietal network; BFT = bilateral frontotemporal network; ↑ = increases; ↓ = decreases; AHRS = Auditory Hallucinations Rating Scale; PANSS = Positive And Negative Syndromes Scale; PSYRATS = Psychotic Symptom Rating Scales; SAPS = Scale For The Assessment of Positive Symptoms; SANS = Scale For The Assessment of Negative Symptoms; VFT = verbal fluency task; ASL = arterial spin labelling; FC = functional connectivity; rCBF = regional cerebral blood flow; CPZE=Chlorpromazine equivalents.

**References:**

1. Slotema CW, Blom JD, de Weijer AD, Diederen KM, Goekoop R, Looijestijn J, Daalman K, Rijkaart A-M, Kahn RS, Hoek HW, et al. Can low-frequency repetitive transcranial magnetic stimulation really relieve medication-resistant auditory verbal hallucinations? Negative results from a large randomized controlled trial. *Biological Psychiatry* (2011) **69**:450–456. doi:10.1016/j.biopsych.2010.09.051

2. Paillère-Martinot M-L, Galinowski A, Plaze M, Andoh J, Bartrés-Faz D, Bellivier F, Lefaucheur J-P, Rivière D, Gallarda T, Martinot J-L, et al. Active and placebo transcranial magnetic stimulation effects on external and internal auditory hallucinations of schizophrenia. *Acta Psychiatrica Scandinavica* (2017) **135**:228–238. doi:10.1111/acps.12680

3. Vercammen A, Knegtering H, Liemburg EJ, den Boer JA, Aleman A. Functional connectivity of the temporo-parietal region in schizophrenia: effects of rTMS treatment of auditory hallucinations. *Journal of Psychiatric Research* (2010) **44**:725–731. doi:10.1016/j.jpsychires.2009.12.011

4. Bais L, Liemburg E, Vercammen A, Bruggeman R, Knegtering H, Aleman A. Effects of low frequency rTMS treatment on brain networks for inner speech in patients with schizophrenia and auditory verbal hallucinations. *Progress in Neuro-Psychopharmacology & Biological Psychiatry* (2017) **78**:105–113. doi:10.1016/j.pnpbp.2017.04.017

5. de Weijer AD, Sommer IEC, Lotte Meijering A, Bloemendaal M, Neggers SFW, Daalman K, Boezeman EHJF. High frequency rTMS; a more effective treatment for auditory verbal hallucinations? *Psychiatry Research* (2014) **224**:204–210. doi:10.1016/j.pscychresns.2014.10.007

6. Schönfeldt-Lecuona C, Grön G, Walter H, Büchler N, Wunderlich A, Spitzer M, Herwig U. Stereotaxic rTMS for the treatment of auditory hallucinations in schizophrenia. *Neuroreport* (2004) **15**:1669–1673. doi:10.1097/01.wnr.0000126504.89983.ec

7. Kindler J, Homan P, Jann K, Federspiel A, Flury R, Hauf M, Strik W, Dierks T, Hubl D. Reduced neuronal activity in language-related regions after transcranial magnetic stimulation therapy for auditory verbal hallucinations. *Biological Psychiatry* (2013) **73**:518–524. doi:10.1016/j.biopsych.2012.06.019

8. Maïza O, Hervé P-Y, Etard O, Razafimandimby A, Montagne-Larmurier A, Dollfus S. Impact of Repetitive Transcranial Magnetic Stimulation (rTMS) on Brain Functional Marker of Auditory Hallucinations in Schizophrenia Patients. *Brain Sciences* (2013) **3**:728–743. doi:10.3390/brainsci3020728

9. Briend F, Leroux E, Delcroix N, Razafimandimby A, Etard O, Dollfus S. Impact of rTMS on functional connectivity within the language network in schizophrenia patients with auditory hallucinations. *Schizophrenia Research* (2017) **189**:142–145. doi:10.1016/j.schres.2017.01.049

10. Fitzgerald PB, Sritharan A, Benitez J, Daskalakis ZJ, Jackson G, Kulkarni J, Egan GF. A preliminary fMRI study of the effects on cortical activation of the treatment of refractory auditory hallucinations with rTMS. *Psychiatry Research* (2007) **155**:83–88. doi:10.1016/j.pscychresns.2006.12.011

11. Homan P, Kindler J, Hauf M, Hubl D, Dierks T. Cerebral blood flow identifies responders to transcranial magnetic stimulation in auditory verbal hallucinations. *Translational Psychiatry* (2012) **2**:e189. doi:10.1038/tp.2012.114

12. Sommer IEC, de Weijer AD, Daalman K, Neggers SF, Somers M, Kahn RS, Slotema CW, Blom JD, Hoek HW, Aleman A. Can fMRI-guidance improve the efficacy of rTMS treatment for auditory verbal hallucinations? *Schizophrenia Research* (2007) **93**:406–408. doi:10.1016/j.schres.2007.03.020

13. Montagne-Larmurier A, Etard O, Razafimandimby A, Morello R, Dollfus S. Two-day treatment of auditory hallucinations by high frequency rTMS guided by cerebral imaging: A 6 month follow-up pilot study. *Schizophrenia Research* (2009) **113**:77–83. doi:10.1016/j.schres.2009.05.006

14. Zöllner R, Hübener A-F, Dannlowski U, Kircher T, Sommer J, Zavorotnyy M. Theta-Burst Stimulation for Auditory-Verbal Hallucination in Very-Late-Onset Schizophrenia-Like Psychosis-A Functional Magnetic Resonance Imaging Case Study. *Frontiers in Psychiatry* (2020) **11**:294. doi:10.3389/fpsyt.2020.00294

15. Giesel FL, Mehndiratta A, Hempel A, Hempel E, Kress KR, Essig M, Schröder J. Improvement of auditory hallucinations and reduction of primary auditory area’s activation following TMS. *European Journal of Radiology* (2012) **81**:1273–1275. doi:10.1016/j.ejrad.2011.03.002

16. Jardri R, Pins D, Thomas P. A case of fMRI-guided rTMS treatment of coenesthetic hallucinations. *The American Journal of Psychiatry* (2008) **165**:1490–1491. doi:10.1176/appi.ajp.2008.08040504

17. Jardri R, Lucas B, Delevoye-Turrell Y, Delmaire C, Delion P, Thomas P, Goeb J-L. An 11-year-old boy with drug-resistant schizophrenia treated with temporo-parietal rTMS. *Molecular Psychiatry* (2007) **12**:320. doi:10.1038/sj.mp.4001968

18. Brady RO, Gonsalvez I, Lee I, Öngür D, Seidman LJ, Schmahmann JD, Eack SM, Keshavan MS, Pascual-Leone A, Halko MA. Cerebellar-Prefrontal Network Connectivity and Negative Symptoms in Schizophrenia. *The American Journal of Psychiatry* (2019) **176**:512–520. doi:10.1176/appi.ajp.2018.18040429

19. Basavaraju R, Ithal D, Thanki M, HR A, Thirthalli J, Pascual-Leone A, Halko M, Brady R, Mehta UM, Kesavan M. T79. INTERMITTENT THETA BURST STIMULATION OF CEREBELLAR VERMIS IN SCHIZOPHRENIA: IMPACT ON NEGATIVE SYMPTOMS AND BRAIN CONNECTIVITY. *Schizophr Bull* (2019) **45**:S234. doi:10.1093/schbul/sbz019.359

20. Dlabac-de Lange JJ, Liemburg EJ, Bais L, Renken RJ, Knegtering H, Aleman A. Effect of rTMS on brain activation in schizophrenia with negative symptoms: A proof-of-principle study. *Schizophrenia Research* (2015) **168**:475–482. doi:10.1016/j.schres.2015.06.018

21. Prikryl R, Mikl M, Prikrylova Kucerová H, Ustohal L, Kasparek T, Marecek R, Vrzalova M, Ceskova E, Vanicek J. Does repetitive transcranial magnetic stimulation have a positive effect on working memory and neuronal activation in treatment of negative symptoms of schizophrenia? *Neuro Endocrinology Letters* (2012) **33**:90–97.

22. Guse B, Falkai P, Gruber O, Whalley H, Gibson L, Hasan A, Obst K, Dechent P, McIntosh A, Suchan B, et al. The effect of long-term high frequency repetitive transcranial magnetic stimulation on working memory in schizophrenia and healthy controls—A randomized placebo-controlled, double-blind fMRI study. *Behavioural Brain Research* (2013) **237**:300–307. doi:10.1016/j.bbr.2012.09.034

23. Liemburg EJ, Dlabac-De Lange JJ, Bais L, Knegtering H, Aleman A. Effects of bilateral prefrontal rTMS on brain activation during social-emotional evaluation in schizophrenia: A double-blind, randomized, exploratory study. *Schizophrenia Research* (2018) **202**:210–211. doi:10.1016/j.schres.2018.06.051

24. Jardri R, Delevoye-Turrell Y, Lucas B, Pins D, Bulot V, Delmaire C, Thomas P, Delion P, Goeb J-L. Clinical practice of rTMS reveals a functional dissociation between agency and hallucinations in schizophrenia. *Neuropsychologia* (2009) **47**:132–138. doi:10.1016/j.neuropsychologia.2008.08.006
